# Healthcare service use in paediatric inflammatory bowel disease: a questionnaire on patient and parent care experiences in Germany

**DOI:** 10.1186/s12876-023-03021-w

**Published:** 2023-11-06

**Authors:** Aletta Boerkoel, Luisa Tischler, Kalina Kaul, Heiko Krause, Ulrike Stentzel, Stefan Schumann, Neeltje van den Berg, Jan de Laffolie, CEDNA study group

**Affiliations:** 1 Institute for Community Medicine, University Medicine Greifswald, Greifswald, Germany; 2 General Pediatrics & Pediatric Gastroenterology, Justus-Liebig-University, Giessen, Germany

**Keywords:** Gastroenterology, Health services research, CEDNA-questionnaire, Paediatric inflammatory bowel disease, Patient experience, Parent experience

## Abstract

**Background:**

Paediatric inflammatory bowel disease (PIBD) patients require chronic care over the lifespan. Care for these patients is complex, as it is adapted for childrens’ life stages and changing disease activity. Guideline based care for this patient group recommends a multidisciplinary approach, which includes in addition to paediatric gastroenterologists, nutritional and psychological care services. For PIBD patients, a discrepancy between available guideline-based multidisciplinary care and actual care has been found from the provider side, but to what extent patients experience this is unclear.

**Objectives:**

To identify which healthcare services were used and whether socio-demographic, geographic or disease related factors have an influence on health service utilisation.

**Methods:**

A standardised questionnaire (CEDNA) was distributed amongst parents of children aged 0–17 diagnosed with PIBD and adolescents (aged 12–17) with a PIBD. Items related to health service use were analysed, these included specialist care, additional care services, reachability of services and satisfaction with care. Logistic regression models on additional service use were calculated. Service availability and reachability maps were made.

**Results:**

Data was analysed for 583 parent and 359 adolescent questionnaires. Over half of the respondents had Crohn’s Disease (CD, patients n = 186 parents n = 297). Most patients and parents reported their paediatric gastroenterologist as their main care contact (patients 90.5%; parents 93%). Frequently reported additional services were nutritional counselling (patients 48.6%; parents 42.2%) and psychological support (patients 28.1%; parents 25.1%). Nutritional counselling was more frequently reported by CD patients in both the patient (OR 2.86; 95%CI 1.73–4.70) and parent (OR 3.1; 95%CI 1.42–6.71) sample. Of the patients, 32% reported not using any additional services, which was more likely for patients with an illness duration of less than one year (OR 3.42; 95%CI 1.26–9.24). This was also observed for the parent population (OR 2.23; 95%CI 1.13–4.4). The population-based density of specialised paediatric gastroenterologists was not proportionate to the spatial distribution of patients in Germany, which may have an influence on access.

**Conclusions:**

Parents and children reported highly specialised medical care. Multidisciplinary care offers do not reach the entire patient population. Access to multidisciplinary services needs to be ensured for all affected children.

**Supplementary Information:**

The online version contains supplementary material available at 10.1186/s12876-023-03021-w.

## Introduction

Paediatric inflammatory bowel disease (PIBD) requires multidisciplinary, complex and long-term treatment across the lifespan, that must be adapted to changing phases of disease activity, localisation and age-specific requirements [1]. PIBD encompasses the diagnoses of Crohn’s disease (CD), ulcerative colitis (UC) and unclassified IBD. These illnesses are associated with impaired quality of life, morbidity and mortality, delayed physical development, impaired social life, and mental health distress [1, 2]. Chronic care for this patient group requires intense interdisciplinary exchange and coordination, but also includes involvement and empowerment of patients and their families [3–6].

With the increase in the incidence and prevalence of PIBD in recent years, especially in developed countries [7–9], and the description of a gap between guidelines and real-world medicine [10–12] research on healthcare provision for this patient group has gained traction [13–15]. In German speaking countries, the implementation of multidisciplinary care teams for the treatment of PIBD is mostly coordinated by the GPGE e.V. (Society for Paediatric Gastroenterology and Nutrition). There are 103 GPGE-certified paediatric practices and clinics in Germany, Austria, Switzerland and the Netherlands (data from 2021). The availability of paediatric gastroenterologists and specialised centres varies between regions, leading to regional differences in access to guideline based care, which in turn may lead to a delay in diagnosis [16]. In Germany, the incidence of PIBD is estimated at 800–1500 children and adolescents [17]. Approximately 8000 PIBD patients presented to hospitals in 2021 [18]. This indicates the importance of providing high-quality care to this population.

The results from a questionnaire distributed to paediatric gastroenterology service providers in Germany showed that one-third of the participating children’s hospitals and two-thirds of the major acute hospitals were unable to meet inpatient care needs because of shortages in resources or internal long waiting times (e.g., endoscopy waiting times). Two thirds of providers were unable to offer outpatient appointments due to a lack of financial resources. The authors highlighted the need for future studies to reflect the perspective of affected patients on their care needs [19].

The current analysis aims to identify which healthcare services were used and whether socio-demographic, geographic or disease related factors influenced service utilisation.

## Methods

To gain patients’ and parents’ perspectives, a questionnaire aiming to identify patients’ and parents’ needs for the improvement of health service provision in PIBD was created (CEDNA; CED-BedarfsaNAlyse [IBD- service needs analysis]). This was a cross-sectional study conducted nationwide from 01.10.2021 to 30.04.2022. The questionnaires were designed in cooperation with patients and their families and with non-financial support from the patient organisation DCCV e.V. (Deutsche Morbus Crohn/Colitis Ulcerosa Vereinigung). It was pretested in a clinical setting.

Through convenience sampling PIBD patients aged 12–17 and parents of children and adolescents with PIBD aged 0–17 were invited to complete their respective questionnaires. The questionnaire included multiple choice questions (multiple answers possible), with a free text field available if the presented options were not fitting. The questionnaires were only available in German. They were distributed by certified centres, as well as by the German patient organisation DCCV e.V. Multiple participation of respondents of both the online and the paper version was prevented by pseudonymised personal questionnaire IDs, where it was highly improbable that multiple respondents fit the criteria of the personal questionnaire ID. If duplicates were identified, the most complete response was kept for analysis.

### Statistical analysis

The questionnaires were included for analysis when information for the following four variables were available: age and gender of the patient, their IBD diagnosis and the time since diagnosis. Frequencies were calculated for all variables. From the questionnaire, items were pre-allocated to the healthcare utilisation topic; both the patient and parent questionnaire included: main care contact person for treatment (paediatric gastroenterologist, general practitioner, paediatric general practitioner, adult gastroenterologist, no main care contact person), satisfaction with care, additional health services used by patient (nutritional counselling (incl. dieticians), do not use any treatment or counselling services, psychological support (incl. psychotherapy, psychiatry, other counselling services), physiotherapy, events to inform about the disease, family counselling, offers for stress management, occupational therapy, outpatient care services/home help, advice from pension insurance, offers from support groups, genetic counselling, counselling through health or long-term care insurance, transition programs, sexual counselling). Only included in the parent questionnaire: satisfaction with health insurer, refused treatment reimbursement.

Logistic regression models were calculated for outcome variables that showed discordance (e.g., 60% answered yes, 40% answered no) in answers. For each of these variables three regression models were examined: a model using illness-related factors (patient and parent: diagnosis, comorbidities, time since diagnosis, current phase of illness; parents: disease pattern), a model using geographic factors (patient and parent: German federal state, size of hometown; parents: driving distance to specialist) and a model using sociodemographic factors (patient and parent: family structure, number of siblings; patients: school level; parents: age of parent, relationship to patient, school diploma, further education diploma).

A sensitivity analysis on the use of psychological support was done, in which patients with a reported psychological comorbidity were excluded from the analysis.

The analysed questions and their translations are included in Supplementary Material 1.

The data analysis was conducted using SAS© Software, Version 9.4 and JMP®, version 17.2, both of the SAS System for Windows.

### Geographical methods

To put the results into context of the healthcare system in Germany, maps were created based on information retrieved from national healthcare registries (data in Table 2).

Using the geographic information system software ArcGIS Pro 3.1 (ESRI, Redlands, CA, USA), a bivariate choropleth map was created to show the quantitative relationship between the two variables “the total number of IBD diagnoses for under 18-year olds” as found in the national hospital diagnostic registry [18] and the “total number of inpatient paediatric gastroenterologists” for the German federal states [20]. By combining two different sets of symbols and colours, changes in the relation of one attribute to another can be illustrated. For this, the procedure classifies each variable and assigns a colour to each class. As a method for the classification, “Quantile” with three classes was used, resulting in a 3 × 3 colour matrix. The classes are divided into ‘low’, ‘medium’, and ‘high’. For each variable, each class contains 33% of the sample; ‘low’ the lowest 33% and ‘high’ the top 33%.

Spatial accessibility of GPGE certified centres (data taken from the GPGE e.V. [21] was operationalized by calculating travel times. Such calculations are based on graph theory and network analyses. ArcGIS Pro provides a network analyst toolbox. Thereof, the “Service Area Analysis” was performed, using the ArcGIS Online Network Data Source from ESRI (https://www.arcgis.com/).

### Ethical considerations

Patient information and consent were included in the questionnaires. The study was approved by the ethics committee of the Justus-Liebig University in Giessen (Reference 07/11).

## Results

**Fig. 1:**
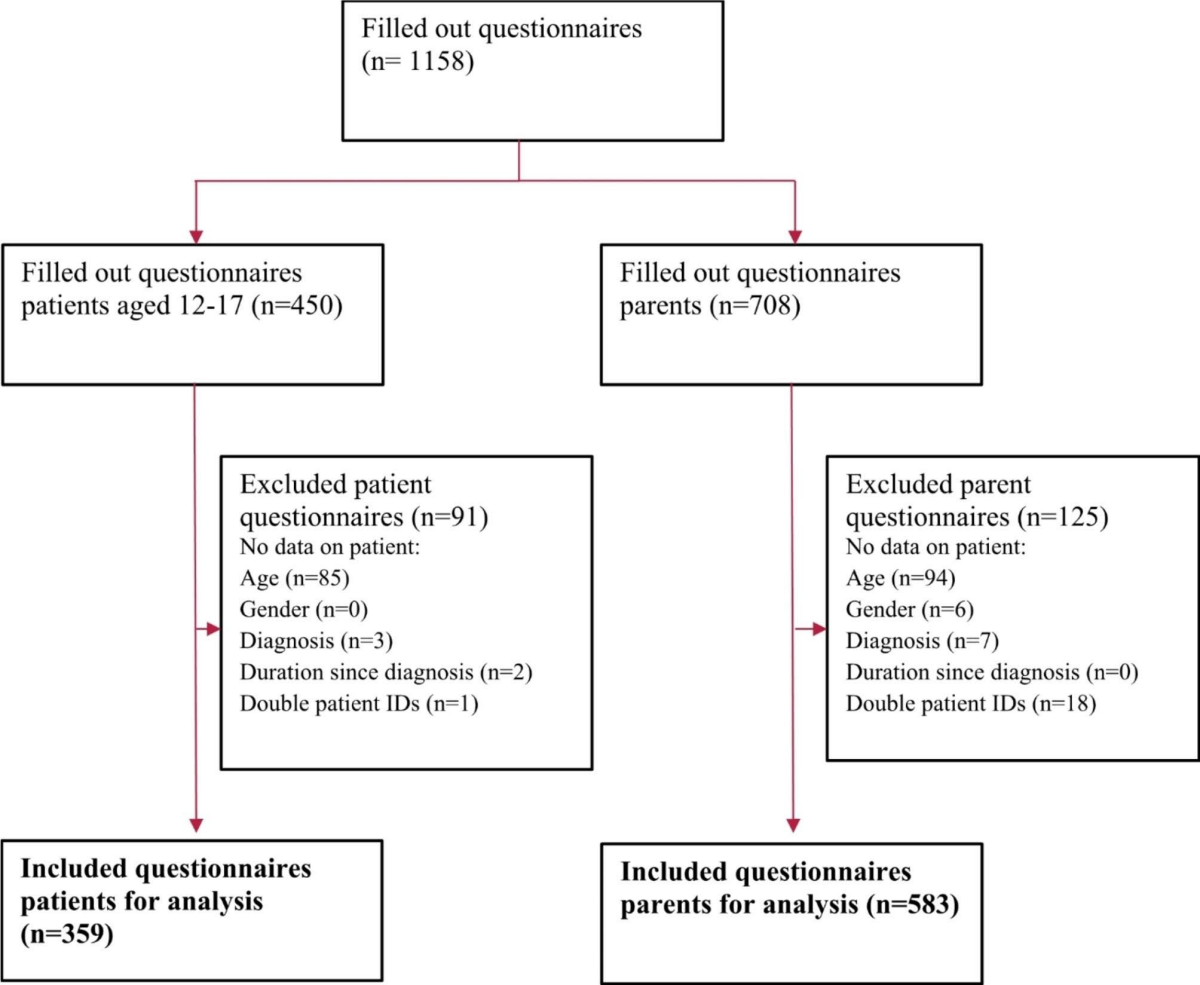
Flow chart showing included and excluded questionnaire for the patients and parents

Figure 1 shows the data flow chart for the questionnaires that were included in the analyses. The paper distribution of the questionnaire had a response rate of 27%.

### Results of the patients’ questionnaires

A total of 359 questionnaires were included in the analyses. The mean age of the patients was 14.8 years (95% CI [14.64; 14.95]), 182 participants were girls, and five were diverse. Most patients reported that they had one or two siblings (n = 268; 74,7%). Table 1 reports the illness related information for the patient group, as reported by both patients and parents.

**Table 1:** Frequency report on IBD-diagnoses, duration of illness, the current phase of the illness and comorbidities

	Patients		Parents	
**IBD-Diagnosis**	n	%	n	%
Crohn’s’ disease	186	**51.81%**	297	**50.94%**
Ulcerative Colitis	155	**43.18%**	240	**41.17%**
Unclassified IBD	18	**5.01%**	46	**7.89%**
**Length of time since diagnosis**				
Less than a year	46	**12.78%**	107	**18.35%**
1–2 years	102	**28.33%**	164	**28.13%**
3–4 years	87	**24.17%**	132	**22.64%**
5–6 years	49	**13.61%**	71	**12.18%**
longer than 6 years	76	**21.11%**	109	**18.70%**
**Phase of diagnosis**				
Diagnosis	6	**1.70%**	26	**4.48%**
Active	37	**10.48%**	92	**15.86%**
Remission	272	**77.05%**	395	**68.10%**
Don’t know	38	**10.76%**	67	**11.55%**
Comorbidities*				
Primary sclerosing cholangitis	19		28	
Dermatological Illnesses	39		62	
Eye related issues	6		10	
Gall- and kidney stones	1		0	
Thrombosis	0		2	
Inflammatory joint illnesses	25		39	
Psychological disorders	32		58	

*multiple comorbidities per patient possible

Most patients (n = 325; 90,5%) reported that a paediatric gastroenterologist had been the primary contact in the past 12 months. Overall, 94% (n = 328) were satisfied with the medical care they received. Table 2 shows the geographical distribution of the sample. Subgroup analysis using the German federal states could not be done, as the distribution of responses was too uneven.

**Table 2:** German federal state level response rate, inhabitants, IBD diagnosis, GPGE certified centres and paediatric gastroenterologists

German federal states	Questionnaire responses patients and parents	Inhabitants < 18-years	IBD diagnosis < 18-years	IBD diagnosis < 18-years per 100.000 inhabitants	GPGE certified centres	Outpatient paediatric gastroenterologists	Inpatient paediatric gastroenterologists
Baden-Württemberg	75	1,910,000	956	50.5	9	10	23
Bavaria	110	2,970,000	1043	47.7	21	10	39
Berlin	25	617,623	398	64.8	4	4	9
Brandenburg	31	408,243	348	85.8	6	3	5
Bremen	2	113,543	55	48.7	2	6	7
Hamburg	9	315,870	131	41.6	3	8	14
Hesse	84	1,068,758	479	45	8	9	14
Lower Saxony	52	1,360,000	786	58.5	10	3	6
Mecklenburg Western Pomerania	21	247,445	121	49	2	0	0
Northrhine-Westphalia	147	3,040,000	2032	67.1	12	25	61
Rhineland Palatinate	20	677,582	393	58.3	3	2	6
Saarland	1	147,668	146	99.3	1	0	1
Saxony	24	862,766	423	65.3	3	1	14
Saxony-Anhalt	26	324,718	286	88.4	5	1	6
Schleswig-Holstein	10	475,796	291	61.4	3	4	2
Thuringia	23	320,925	285	88	2	1	5

**Fig. 2:**
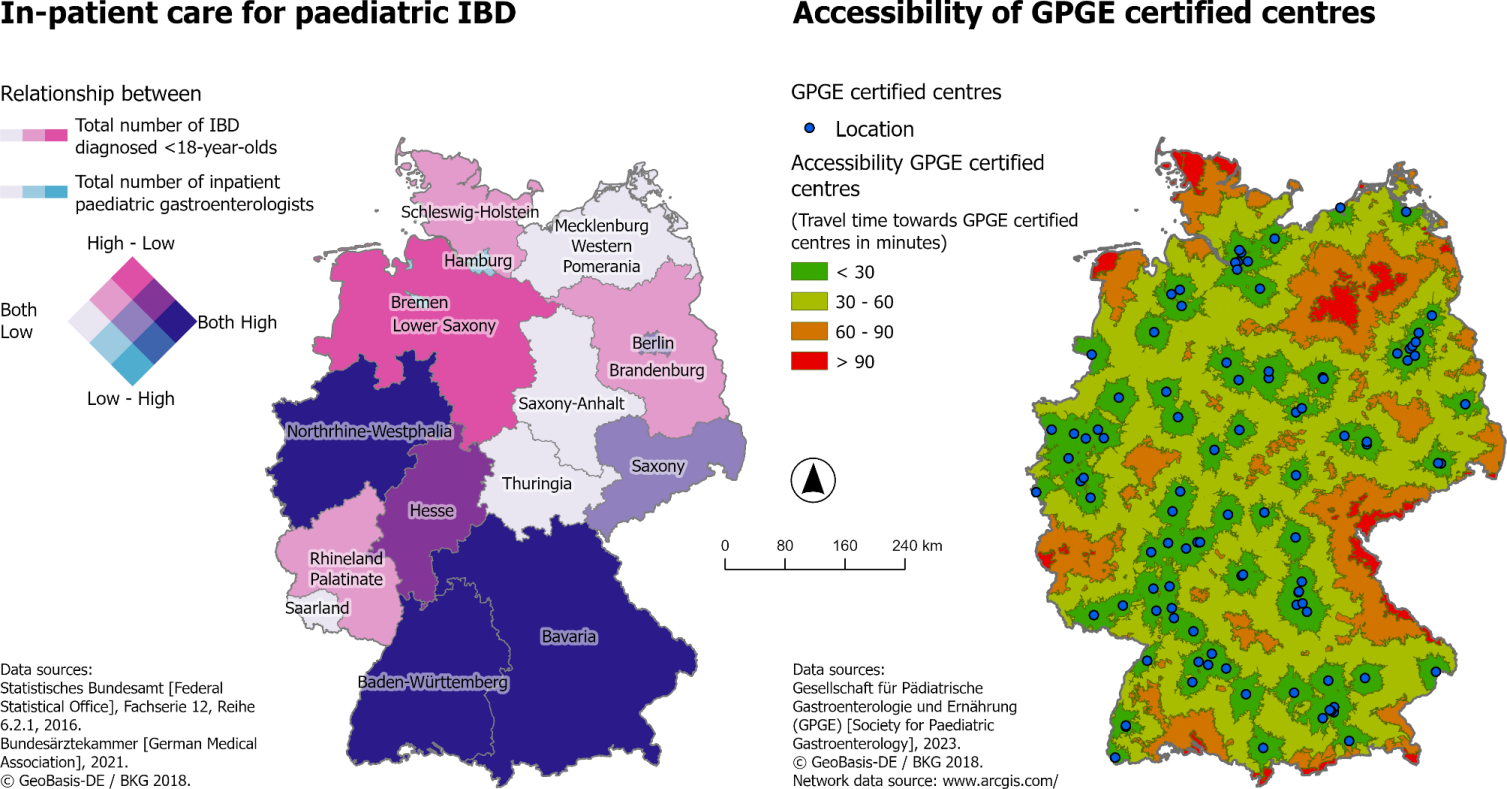
Availability of paediatric IBD care services in Germany related to the number of patients. (a) Relationship between the total number of IBD diagnoses (codes K50-K52 from the International Classification of Diseases-10 [ICD-10; 1] for the under 18 years old as found in the national hospital diagnostic registry and the total number of inpatient paediatric gastroenterologists per German state. Dark blue indicates a high number of both patients and specialists, grey indicates a low number of both. Pink indicates a high number of patients and a low number of specialists. Light blue indicates a high number of specialists and a low number of patients. (b) Approximate driving time to GPGE certified treatment centres

*Data from 2021. Retrieved from www.gbe-bund.de 18, 20. Note: Data was used for the creation of Fig. 2.

Nutritional counselling (n = 171; 48.6%) was the most commonly used additional health service, followed by psychological support (n = 99; 28.1%) and physiotherapy (n = 54; 15.3%). Approximately one-third of patients (n = 114; 32.4%) reported not using or having used any additional services at all. A complete overview of responses to additional service use can be found in Supplementary Material 2.

**Table 3:** Multivariate logistic regression models of patient responses on additional service use

	Nutritional counselling		Psychological services		No additional services	
	Odds Ratio	95% Confidence Interval	Odds Ratio	95% Confidence Interval	Odds Ratio	95% Confidence Interval
**Soziodemographic model**						
**Man**	0.81	0.52–1.24	0.62	0.38–1.01	1.03	0.65–1.62
**Age of patient**	0.86*	0.72–0.99	1.00	0.85–1.18	1.11	0.94–1.31
**Family structure**						
two caretakers	1.46	0.30–7.09	0.18*	0.04–0.78	0.69	0.10–4.57
one caretaker	1.06	0.19–5.88	0.56	0.10–2.75	1.05	0.19–5.93
**Siblings**	1.01	0.55–1.78	1.06	0.56–2.07	0.93	0.19–5.93
**School type**						
Primary school	0.49	0.02–10.27	^	^	3.01	0.14–65.39
Mittel-/Ober-/Realschule	0.70	0.22–2.24	^	^	1.30	0.38–4.42
Gesamtschule	0.57	0.17–1.99	^	^	0.97	0.26–3.65
Gymnasium	0.79	0.26–2.36	^	^	1.14	0.36–3.61
**Illness related factors**						
**Man**	0.93	0.57–1.57	0.82	0.48–1.39	0.97	0.58–1.63
**Age of patient**	0.87	0.73–1.02	0.97	0.81–1.17	1.07	0.89–1.28
**Diagnosis**						
Morbus Crohn	0.79	0.0.25–2.46	0.75	0.23–2.46	1.04	0.31–3.56
Ulceritive Colitis	0.28***	0.09–0.88	1.12	0.34–3.72	1.62	0.47–5.59
**Comorbidities**	0.68	0.28–1.62	18.34***	6.61–50.90	0.13**	0.03–0.59
**Time since diagnosis**						
less than 1 year	0.60*	0.25–1.47	0.47	0.16–1.36	2.34	0.94–5.78
1–2 years	1.30	0.64–2.65	0.76	0.35–1.66	1.41	0.67–2.99
3–4 years	1.52	0.75–3.08	1.36	0.64–2.91	0.83	0.38–1.79
5–6 years	1.60	0.70–3.62	1.62	0.69–3.82	0.68	0.27–1.72
**Phase of illness**						
Diagnosis	2.07	0.35–12.44	^	^	2.87	0.49–16.82
Active	0.61	0.28–1.36	^	^	1.37	0.61–3.05
**Geographical factors**						
**Number of people living in hometown**						
more than 500.000	1.07	0.52–2.22	1.16	0.53–2.53	0.83	0.38–1.81
100.000 -500.000	0.55	0.26–1.12	0.90	0.39–2.07	0.64	0.27–1.48
20.000-100.000	0.61	0.33–1.14	1.03	0.53–2.01	1.00	0.53–1.91
5000-20.000	1.03	0.59–1.79	0.68	0.36–1.28	1.01	0.60–1.92

*p < 0.05; ** p < 0.01; ***p < 0.001; ^ excluded from analysis because of complete separation of data-set

Note: reference values for categorical variables were: other family structure, no siblings, no longer in school, unclassified IBD, more than 6 years since diagnosis, remission, the disease is becoming more active and symptom intensity varies, less than 5000 people living in hometown.

The complete results of the logistic regression models for the patient responses are reported in Table 3. The logistic regression models run on the outcome “reported use of nutritional counselling”, yielded significant results for the model with illness-related factors. Patients with CD were more likely to use nutritional services (OR 2.86; 95% CI 1.73–4.70) than UC patients. Patients diagnosed 3–4 years ago (OR 2.53 95% CI 1.08–5.97) and those diagnosed 5–6 years ago (OR 2.66; 95% CI 1.02–6.91) were more likely to use nutritional counselling than those in their first year after diagnosis.

Logistic regression models run on the outcome “reported use of psychological services” yielded significant results for the models with disease-related and sociodemographic factors. Paediatric patients with IBD who also suffer from a comorbidity make significantly more use of psychological services (OR 18.34; 95% CI 6.61–50.90). Furthermore, the family structure significantly influences the use of psychological services. Children in single parents’ households are more likely to receive psychological services than those who live with both parents (OR 3.09; 95% CI 1.47–6.54). Additionally, children who live with anyone other than their parent(s) are more likely to use psychological services than those who live with two parents (OR 5.51; 95% CI 1.28–28.18).

For a sensitivity analysis, all children that reported a psychological comorbidity were excluded (n = 38), this resulted in a no longer significant explanatory factor of comorbidity on the use of psychological services.

The outcome “not using any additional services” was best explained by the illness related- model. Those who reported not having any comorbidities were more likely not to use additional services (OR 7.55 95% CI 1.69–33.76) than those with. Patients who were diagnosed less than one year ago were more likely not to use services compared to patients with a diagnosis 5–6 years ago (OR 3.42 95% CI 1.26–9.24).

### Results of the parents’ questionnaires

A total of 583 questionnaires were included in the analysis. The average age of the children of the participating parents was 12.9 years (95% CI [12.63; 13.18]). 287 were girls, and none were diverse. Most parents reported that their children had one or two siblings (71.3%). Table 2 shows the geographical distribution of the sample. Subgroup analysis using the German federal states could not be done, as the distribution of responses was too uneven.

Almost all parents reported that their children received health services from a paediatric gastroenterologist (99.1%), who was also the main point of contact in 93% of cases. The 41 people who indicated having a different main contact person, stated that this was their paediatrician (14 responses), the adult gastroenterologist (4 responses) or their general practitioner (2 responses). The remaining responses did not give an indication of a main contact person. The additional care services that were used were nutritional counselling (n = 233; 42.4%), psychological support (n = 138; 25.1%) and physiotherapy (n = 53; 9.7%). Just over a third of parents indicated that their children had never used additional healthcare services (n = 204; 37.2%). A third of respondents indicated using multiple services (n = 168, 28.8%); of these 69 respondents use both nutritional and psychological support.

The logistic regression models run on the reported use of nutritional counselling, yielded significant results for the model with illness-related factors. This model explained service use differences better than the models including sociodemographic or geographical factors. The model indicated that patients with CD were more likely (OR 3.1; 95% CI 1.42–6.71) to seek nutritional counselling compared to patients with unclassified IBD.

The complete results of the logistic regression models for the parent responses are reported in Table 4. The use of psychological services was explained by both the sociodemographic and illness- related factors model. With every increased year of age, patients were more likely to seek services (OR 1.15; 95% CI 1.06–1.25), and male patients were less likely than female patients to seek services (OR 0.65; 95% CI 0.43–0.99). The presence of comorbidities decreased the likelihood of seeking psychological services (OR 0.37; 95% CI 0.23–0.59). When patients with a psychological comorbidity were excluded (n = 53), comorbidities were no longer significantly explaining psychological counselling, but no changes in significance were found for the duration since diagnosis. Patients diagnosed with IBD in the past year were less likely to seek psychological services than patients with a diagnosis of more than 6 years ago (OR 0.34; 95% CI 0.13–0.85).

**Table 4:** Multivariate logistic regression models for parent responses on additional service use

	Nutritional counselling		Psychological services		No additional services	
	Odds Ratio	95% Confidence Interval	Odds Ratio	95% Confidence Interval	Odds Ratio	95% Confidence Interval
**Soziodemographic model**						
**Man**	1.26	0.88–1.80	0.66*	0.43-1.00	1.20	0.83–1.74
**Age of patient**	1.01	0.95–1.08	1.12**	1.03–1.21	0.93*	0.88–0.99
**Parent younger than 40**	1.03	0.64–1.66	0.82	0.46–1.46	0.59*	0.35–0.97
**Familial relationship**						
Mother	0.23	0.05–1.18	0.84	0.16–4.52	1.00	0.20–4.86
Father	0.23	0.04–1.22	0.41	0.07–2.47	1.29	0.25–6.76
**Family structure**						
two caretakers	1.06	0.35–3.26	0.80	0.21–3.12	0.46	0.15–1.42
one caretaker	0.86	0.26–2.91	1.83	0.44–7.63	0.50	0.15–1.67
**Siblings**						
None	0.89	0.43–1.85	0.50	0.21–1.16	1.40	0.63–3.10
1–2	0.76	0.40–1.44	0.64	0.32–1.30	1.50	0.75–3.01
**School diploma**						
No school diploma	1.02	0.19–5.60	0.76	0.09–6.20	3.39	0.59–19.46
Hauptschulabschluss	2.52	0.96–6.62	1.64	0.59–4.55	1.23	0.44–3.44
Realschule	1.07	0.66–1.76	0.79	0.45–1.39	1.72	1.03–2.89
**Further education diploma**						
No professional diploma	0.89	0.27–2.91	1.15	0.28–4.71	0.69	0.19–2.43
Professional diploma	0.84	0.48–1.49	1.26	0.65–2.45	0.76	0.42–1.38
University of applied sciences	1.09	0.63–1.90	1.60	0.84–3.05	0.82	0.45–1.46
**Illness related factors**						
**Man**	1.19	0.80–1.76	0.67	0.42–1.05	1.30	0.87–1.95
**Age of patient**	0.98	0.98–1.04	1.15**	1.06–1.25	0.99	0.93–1.05
**Diagnosis**						
Morbus Crohn	3.09***	1.42–6.71	1.56	0.60–4.01	0.42**	0.19–0.89
Ulceritive Colitis	0.90**	0.41-2.00	1.15	0.44-3.00	0.88	0.41–1.90
**Comorbidities**	1.17	0.77–1.78	0.37***	0.23–0.59	1.49	0.96–2.30
**Time since diagnosis**						
less than 1 year	0.87	0.44–1.71	0.337**	0.13–0.85	2.2**	1.13–4.42
1–2 years	1.37	0.77–2.45	0.82	0.43–1.58	1.51	0.84–2.72
3–4 years	1.29	0.71–2.36	1.334*	0.69–2.57	0.92	0.49–1.71
5–6 years	1.77	0.88–3.53	1.02	0.48–2.18	0.63*	0.30–1.37
**Phase of illness**						
Diagnosis	0.32	0.10-1.00	1.53	0.41–5.69	2.45	0.87–6.86
Active	0.74	0.39–1.38	1.25	0.63–2.50	1.24	0.66–2.35
**Disease activity pattern**						
Active disease followed by sustained remission	0.80	0.22–2.93	0.23	0.06–0.91	6.87*	1.30-36.29
There are repeated episodes of illness interrupted by periods of inactivity.	0.86	0.24–3.07	0.27	0.07–1.03	4.65	0.90-24.07
The disease is constantly active, the symptoms vary in severity.	0.99	0.28–3.53	0.32	0.08–1.22	4.52	0.88–23.25
**Geographical factors**						
**Number of people living in hometown**						
more than 500.000	1.28	0.69–2.37	0.91	0.46–1.79	0.97	0.50–1.86
100.000 -500.000	0.63	0.33–1.21	0.67	0.33–1.38	1.52	0.80–2.89
20.000-100.000	0.91	0.55–1.50	0.59	0.33–1.05	1.31	0.79–2.19
5000-20.000	1.14	0.72–1.80	0.586	0.35-1.00	1.15	0.71–1.86
**Driving time to paediatric gastroenterologist**						
Less than 30 min	1.03	0.60–1.77	0.938	0.52–1.71	1.14	0.65–1.98
30–60 min	1.12	0.70–1.79	0.77	0.46–1.30	1.11	0.68–1.80

*p < 0.05; ** p < 0.01; ***p < 0.001

Note: reference values for categorical variables were: other familial relationship (e.g. grandparents, carer, etc.), parent younger than 40, other family structure, three or more siblings, gymnasium, university diploma, unclassified IBD, more than 6 years since diagnosis, remission, the disease is becoming more active and symptom intensity varies, less than 5000 people living in hometown, more than 60 min driving time.

Not using any additional healthcare services was explained by illness related factors. CD patients were less likely to not use services (OR 0.41; 95% CI 0.19–0.89), patients who were diagnosed in the past year were more likely to not use services (OR 2.23; 95% CI 1.13–4.4), but those who were diagnosed 5–6 years ago were less likely to not use services (OR 0.63; 95% CI 0.30–1.37). Patients with an active disease that was followed by a sustained remission were more likely not to use additional services compared to patients who have an illness that is getting more active (OR 6.8; 95% CI 1.30-36.29).

With the coverage of health insurance, 16.3% of the parents were dissatisfied and 8.51% stated that they had been refused treatment in the past. Among the group of dissatisfied parents, their children were characterised by a more unstable course of the disease (n = 53 out of N = 90). Most of these paediatric patients received their IBD diagnosis in the 4 years before the questionnaire was distributed (n = 63 out of N = 91).

### Geographical analysis

The distribution of responses over the German states was in line with the population of children and adolescents under 18 years old living in these states (see Table 2; Fig. 2a). The majority of responses originated from the states of Nordrhine Westfalia, Bavaria, and Baden Württemberg, which is reflected in the higher number of GPGE-certified centres in these states (see Fig. 2b). However, the highest prevalence of PIBD is in Saarland, Thuringia, Saxony-Anhalt, and Brandenburg, thus indicating an inability to effectively reach the PIBD population [18].

In Lower Saxony, there is a high number of individuals under 18 years old diagnosed with IBD but a relatively low number of certified paediatric gastroenterological specialists (see Fig. 2a). In the northwest of Lower Saxony, the driving time to a specialist centre is 60 min or longer (see Fig. 2a). In the state with the largest population of under 18 years olds, Northrhine-Westphalia, there is a high number of IBD diagnoses in that population and a high number of available gastroenterological specialists. The certified centres are reached by car in less than an hour, and, in most cases, in less than 30 min. In Mecklenburg-Western Pomerania, Schleswig-Holstein and Brandenburg, a sizeable area has a driving distance of more than an hour. These states also have a lower number of inhabitants, fewer patients with IBD and fewer specialists.

## Discussion

The CEDNA questionnaire provided insights into the self-reported healthcare service use of children and adolescents with IBD. Respondents reported a very high satisfaction with their PIBD care, despite previously identified shortages in supply [19]. Supporting this, almost all patients and parents had a paediatric gastroenterologist as their main point of treatment contact. Because IBD presents differently in children and adolescents than in adults, the guidelines recommend that this patient population should be treated by a paediatrician with training in paediatric gastroenterology [5, 6, 22, 23]. The current findings suggest that, at least in the current study population, shortages in care provision are either not perceived by patients and their families, or are already taken for granted and excluded from evaluation of satisfaction with care. Another point would be the selected cohort of patients through the applied distribution channels of the survey (see also the limitations part), so that some less connected patients without paediatric gastroenterologist care in a reachable distance may not have had access to the study. This is supported by the analysis of epidemiological data and the population below 18 years of age in different regions of Germany.

As with most chronic illnesses, a multidisciplinary approach is considered a better indicator of high-quality care [24]. Multidisciplinary care has been found to result in less frequent presentation of IBD patients to outpatient and inpatient facilities [25]. The additional services reported by patients in the current paper show an underutilisation of multidisciplinary care. The most commonly used service was nutritional counselling, but less than half of the respondents indicated its usage. A further reason for the underutilisation of nutritional counselling in Germany is related to reimbursement practice. Nutritional therapy is part of the initial therapy after diagnosis and is provided by a dietary professional as part of the multi-professional team. Follow-up care with a dietary professional is however, often not reimbursed by the health insurer. This contrasts the shown usefulness of follow-up care with a nutritional specialist for the treatment of PIBD [26]. To aid better care for patients, services ought to be reimbursed, or new ways of providing education, such as online resources or apps need to be developed and implemented into care. Scientific societies such as the local GPGE network and the European ESPGHAN could take up a more active role in this.

Nutritional counselling was more likely to be used by patients with Crohn’s disease, which might in part be due to the role nutrition plays in remission induction and maintenance therapy [5, 27]. The current developments in nutritional therapy, including Crohn’s Disease Exclusion Diet (CDED) could have led to a time effect in the current responses. This can only be clarified in follow-up observations.

Additionally, patients with diagnoses that date further back were more likely to seek nutritional counselling. With an increased duration of one’s illness, knowledge about the illness and its treatment increase, therefore these patients could have been exposed to more information on accessing services. This idea was confirmed by the increased likelihood of not using services in the first year since diagnosis.

The use of psychological services was reported by approximately one third of respondents. Interestingly, there were contradictory results between the answers of the patients and the parents. Patients with comorbidities were more likely to report using services, however, parents of children with comorbidities were less likely to report using psychological care services. This likely originates from the sample, as more parents responded to the questionnaire than children, however, it is something to be investigated further. Logically an increased disease burden could lead to more mental health problems, thus making the results of the parent questionnaire unexpected. On the other hand, a more organ-centred somatic view of parents in families with comorbidities could lead to severe underutilisation of psychological services or underreporting of its usage, because the somatic aspects of disease are mainly recognised as relevant. The current developments towards destigmatising mental health problems in the younger generations could also contribute to the discrepancy, as patients might be more open to reporting on their mental health state than their parents might be [28].

The use of psychological support was, along with comorbidities, partly explained by the family living situation. Children living in two parent households were less likely to seek psychological help than their peers who grew up in single-parent homes. The literature in the general population is split on the use of mental health services and family structure; on the one hand studies show that children and adolescents growing up in single parent households have more mental health issues, on the other hand, it is also reported that they are less likely to seek mental health services [29, 30]. Based on our sensitivity analysis, which excluded patients with a comorbid psychological diagnosis, it was found that in the both the patient and the parent sample comorbidities no longer explained psychological service use This could indicate that psychological service use is largely explained by psychological comorbidities. This should be investigated in future research, as a need for psychological care for PIBD patients has been confirmed in the literature [31–33]. For example, Engelmann et al. [34] showed in a PIBD sample that over 50% of included patients met the Diagnostic and Statistical Manual of Mental Disorders-IV criteria for one or more psychiatric disorders. Similarly, Weber et al. [35] identified that 25% of PIBD patients show latent or manifest depressive symptoms. Adolescents with IBD have a higher ratio of depressive and internalising disorders compared to their peers with other chronic illnesses. Therefore, it is important to strengthen screening of mental health problems and, when needed, referral to psychological services for the PIBD population.

Further relationships between service utilisation and sociodemographic factors could not be identified. Past research has looked at associations between healthcare service use and sociodemographic factors, albeit only for primary and urgent care. Service use was related to the education level of the parent, with a lower level of education associated with an increased use of emergency services [14]. In our sample, this finding could not be replicated. Comparable studies looking at factors influencing health service utilisation in PIBD are limited and focused on the USA, making comparisons to a German population difficult [15].

The distribution of inpatient PIBD patients and availability of paediatric gastroenterologist on the level of individual German states indicated that the regions of Schleswig-Holstein, Lower Saxony and the Rhineland-Palatinate have more patients than physicians compared to other states in Germany. However, from Fig. 2a it can also be read that the city states of Bremen and Hamburg (encapsulated by Schleswig-Holstein and Lower-Saxony) have more paediatric gastroenterologist per patient than the rest of the German states. It can be assumed that the city states contribute to the care provision for the surrounding states. Only in the area of Berlin-Brandenburg this seems more unlikely, as Berlin does not have a large availability of paediatric gastroenterologists.

The travel time to the paediatric gastroenterologist was longer than 30 min for most of Germany (see Fig. 2b), which was also indicated by parents as the needed travel time to their paediatric gastroenterologist. The long driving times in these German regions could be seen as especially burdensome, as PIBD patients have several doctor visits scheduled each year. Some regions have few patients, and thus fewer doctors and longer travel times. For these regions, it is important that solutions are found to care for the few that need specialist care, for example, through e-health solutions. Several studies and reviews have identified that a shorter travel time to providers is preferred by patients and leads to better health outcomes and access to care [36–41]. The link between satisfaction with care and travel time for a German population should be investigated to determine the spatial placement of specialist paediatric gastroenterologists.

### Limitations

The present analysis has some limitations. Due to the irregular distribution of the response rates at the level of the federal states, no statement using logistic regression models could be made about regional differences in the use of healthcare services. Based on the number of IBD cases per state (Fig. 2a; Table 3.), a higher response rate would have been expected in some states, such as Lower Saxony and Saarland. The survey was mainly distributed across GPGE-certified clinics and DCCV, so regions with fewer or no GPGE-certified clinics are underrepresented and patients without access to specialized care are also less likely to be organized in patient organisations. Therefore, we cannot make any statements about the use of healthcare services by paediatric patients who receive medical care from their paediatrician or family physician. This could be assumed to be the case, especially in rural areas with poorer health care infrastructures, where no paediatric gastroenterology centre is reachable.

As the current analyses are based on self-reported use of healthcare services, it is not a true reflection of service use of this population in Germany. However, based on the recruitment through specialised centres and the close relationship of PIBD families to their care team, the results are more likely to overestimate the level of perceived care provision.

## Conclusion

Based on self-reported health service utilisation, service provision for PIBD patients in Germany is medically highly specialised, but the use of multidisciplinary services, such as guideline recommended nutritional and psychological support services needs to be strengthened.

### Electronic supplementary material

Below is the link to the electronic supplementary material.

Supplementary Material 1

Supplementary Material 2

## Data Availability

Data can be made available upon reasonable request through the corresponding author.
