# Supplementary material for: Healthcare service use in paediatric inflammatory bowel disease: a questionnaire on patient and parent care experiences in Germany
Source: BMC Gastroenterol. 2023 Nov 6;23:378. doi: 10.1186/s12876-023-03021-w (PMC10626645; doi:10.1186/s12876-023-03021-w)
Supplement: Supplementary file 1 — Supplementary Material 1 [file 12876_2023_3021_MOESM1_ESM.docx]

Additional Material 1.

**
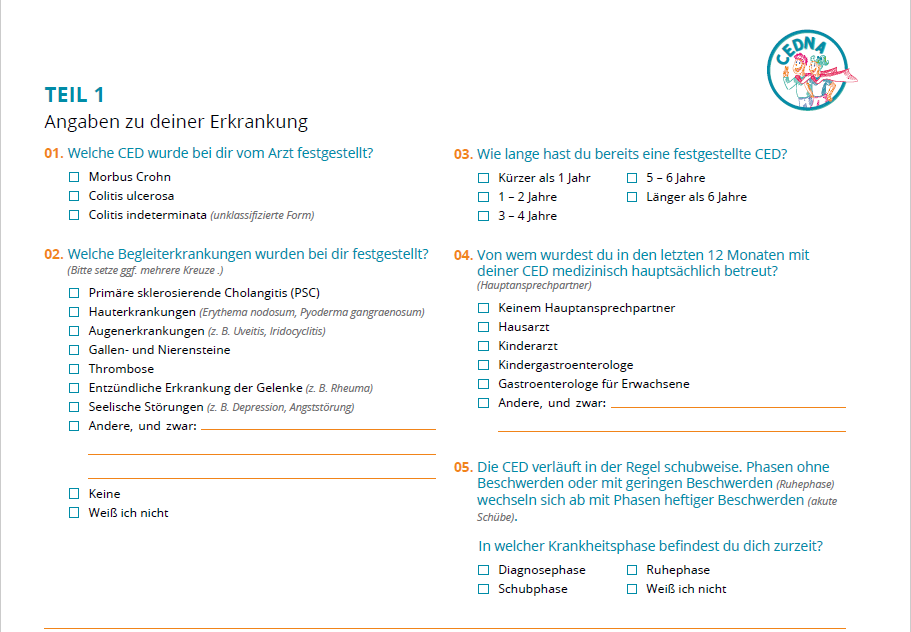
Analysed questions of the German questionnaire for 12–17-year-olds and their translations to English.**

PART 1

Information about your condition

1. Which IBD has been diagnosed by your doctor?

- Crohn's disease
- Ulcerative colitis
- Indeterminate colitis (unclassified form)

1. Which coexisting conditions have been diagnosed in your case? (If applicable, check multiple boxes.)

- Primary sclerosing cholangitis (PSC)
- Skin conditions (e.g., Erythema nodosum, Pyoderma gangraenosum)
- Eye conditions (e.g., Uveitis, Iridocyclitis)
- Gallstones and kidney stones
- Thrombosis
- Inflammatory joint disease (e.g., Rheumatoid arthritis)
- Mental health disorders (e.g., Depression, Anxiety disorder)
- Other, please specify: …..
- None
- I don't know

1. How long have you had a confirmed diagnosis of IBD?

- Less than 1 year
- 1-2 years
- 3-4 years
- 5-6 years
- More than 6 years

1. Who has primarily provided medical care for your IBD in the last 12 months? (Main point of contact)

- No main point of contact
- General practitioner (Hausarzt)
- Pediatrician
- Pediatric gastroenterologist
- Adult gastroenterologist
- Other, please specify: ….

1. IBD typically has a relapsing and remitting course. Phases with no symptoms or mild symptoms (remission phase) alternate with phases of severe symptoms (acute flares). Which phase of the disease are you currently in?

- Diagnosis phase
- Remission phase
- Flare-up phase
- I don't know


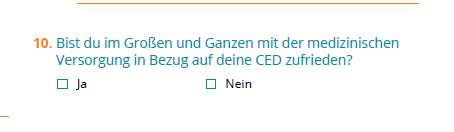


10. Are you generally satisfied with the medical care you receive for your IBD?

1. Yes
2. No


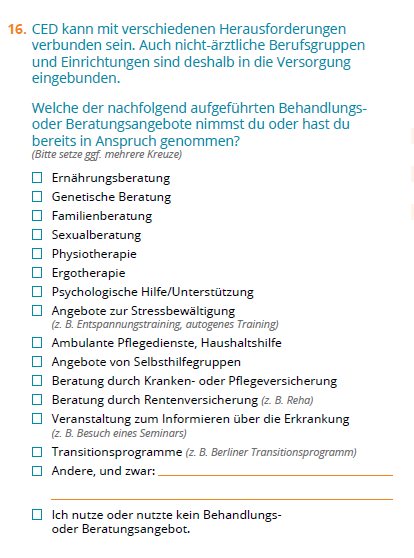


16. CED can be associated with various challenges, and non-medical professionals and facilities are involved in the care as well.

Which of the following treatment or counselling services have you used or have you already used? (Please check multiple boxes if applicable.)

- Nutritional counselling
- Genetic counselling
- Family counselling
- Sexual counselling
- Physiotherapy
- Occupational therapy
- Psychological help/support
- Stress management programs (e.g., relaxation training, autogenic training)
- Outpatient nursing services, home help
- Services from support groups
- Counselling from health or long-term care insurance
- Counselling from pension insurance (e.g., rehabilitation)
- Events to inform about the condition (e.g., attending a seminar)
- Transition programs (e.g., Berlin Transition Program)
- Other, please specify:
-
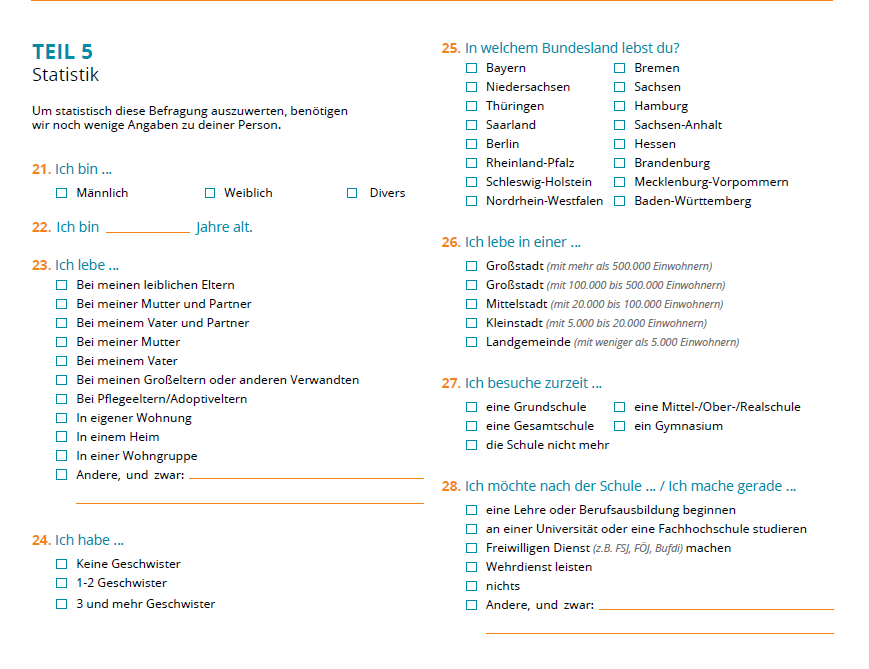
I do not use or have not used any treatment or counselling services

PART 5 Statistics

To statistically evaluate this survey, we need a few more details about you.

1. I am...

- Male
- Female
- Non-binary / Diverse

1. I am … years old.
2. I live...
3. With my biological parents
4. With my mother and partner
5. With my father and partner
6. With my mother
7. With my father
8. With my grandparents or other relatives
9. With foster/adoptive parents
10. In my own apartment
11. In a care facility
12. In a group home
13. Other, please specify: …
14. I have...
15. No siblings
16. 1-2 siblings
17. 3 or more siblings
18. In which federal state (Bundesland) do you live?

- Bavaria
- Bremen
- Lower Saxony
- Saxony
- Thuringia
- Hamburg
- Saarland
- Saxony-Anhalt
- Berlin
- Hesse
- Rhineland-Palatinate
- Brandenburg
- Schleswig-Holstein
- Mecklenburg-Western Pomerania
- North Rhine-Westphalia
- Baden-Württemberg

1. I live in a...

- Major city (with more than 500,000 residents)
- Major city (with 100,000 to 500,000 residents)
- Mid-sized city (with 20,000 to 100,000 residents)
- Small town (with 5,000 to 20,000 residents)
- Rural area (with fewer than 5,000 residents)

1. I currently attend...

- An elementary school
- A middle/high school
- A comprehensive school
- A gymnasium (academic high school)
- I am no longer in school

1. After school, I want to / I am currently...

- Begin an apprenticeship or vocational training
- Study at a university or college (Fachhochschule)
- Engage in voluntary service (e.g., FSJ, FÖJ, Bufdi)
- Complete military service
- Do nothing
- Other, please specify:

**
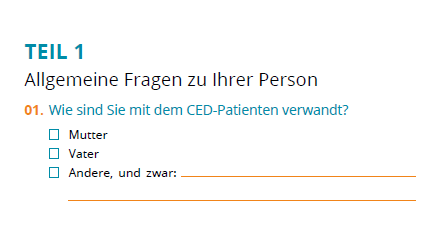
Analysed questions of the German questionnaire for parents and their translations to English.**

1. How are you related to the IBD (Inflammatory Bowel Disease) patient?

- Mother
- Father
-
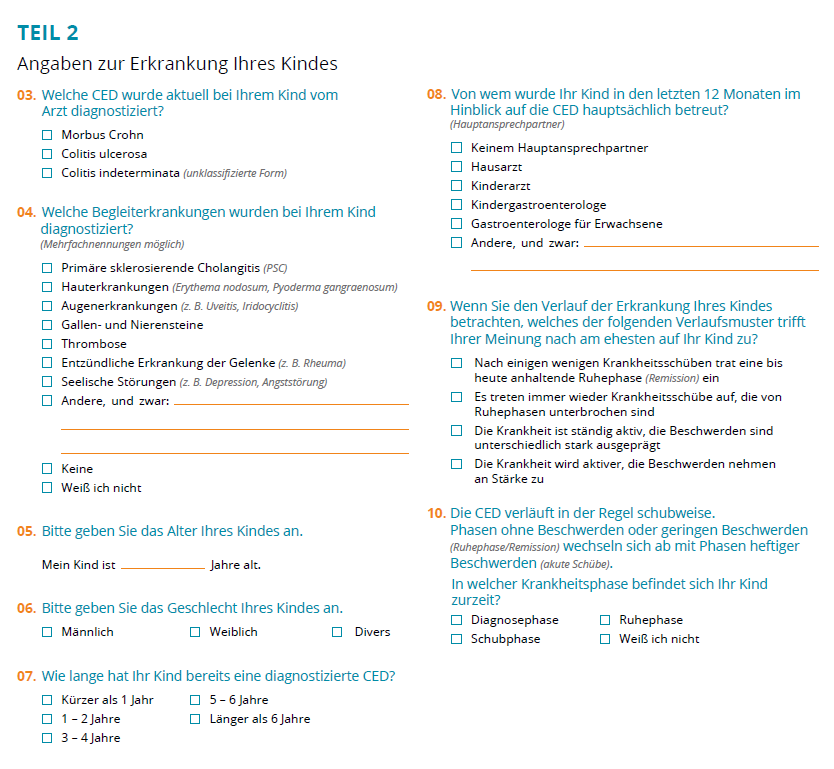
Other, please specify: …

PART 2

Information about your child's condition

1. Which IBD has currently been diagnosed in your child by the doctor?

- Crohn's disease
- Ulcerative colitis
- Indeterminate colitis (unclassified form)

1. Which coexisting conditions have been diagnosed in your child's case? (Select multiple if applicable.)

- Primary sclerosing cholangitis (PSC)
- Skin conditions (e.g., Erythema nodosum, Pyoderma gangraenosum)
- Eye conditions (e.g., Uveitis, Iridocyclitis)
- Gallstones and kidney stones
- Thrombosis
- Inflammatory joint disease (e.g., Rheumatoid arthritis)
- Mental health disorders (e.g., Depression, Anxiety disorder)
- Other, please specify:
- None
- I don't know

1. Please indicate your child's age.

My child is … years old.

1. Please specify your child's gender.

- Male
- Female
- Non-binary / Diverse

1. How long has your child had a diagnosed IBD?

- Less than 1 year
- 1-2 years
- 3-4 years
- 5-6 years
- More than 6 years

1. Who has primarily provided medical care for your child's IBD in the last 12 months? (Main point of contact)

- No main point of contact
- General practitioner (Hausarzt)
- Pediatrician
- Pediatric gastroenterologist
- Adult gastroenterologist
- Other, please specify:

1. When considering the course of your child's illness, which of the following patterns of progression do you believe best describes your child's situation?

- After a few disease flares, a sustained remission phase (remission) has occurred to this day.
- There are recurring disease flares interrupted by remission phases.
- The disease is constantly active, and symptoms vary in severity.
- The disease is becoming more active, and symptoms are increasing in intensity.

1. IBD typically has a relapsing and remitting course. Phases with no symptoms or mild symptoms (remission) alternate with phases of severe symptoms (acute flares). In which phase of the disease is your child currently?

- Diagnosis phase
- Remission phase
- Flare-up phase
-
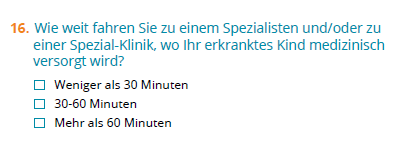
I don't know

16. How far do you travel to a specialist and/or a specialized clinic for the medical care of your ill child?

- Less than 30 minutes
- 30-60 minutes
-
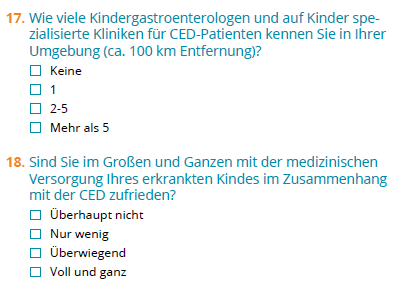
More than 60 minutes

1. How many pediatric gastroenterologists and clinics specialized in treating CED patients for children do you know in your area (approximately within 100 km)?

- None
- 1
- 2-5
- More than 5

1. Are you generally satisfied with the medical care your ill child receives in connection with CED?

- Not at all
- Slightly
- Mostly
-
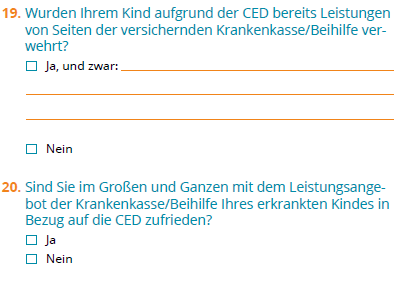
Completely

1. Has your child been denied benefits by the health insurance or assistance program due to CED?

- Yes, namely:
- No

1. Are you generally satisfied with the services provided by your ill child's health insurance or assistance program concerning CED?

- Yes
- No
  1.
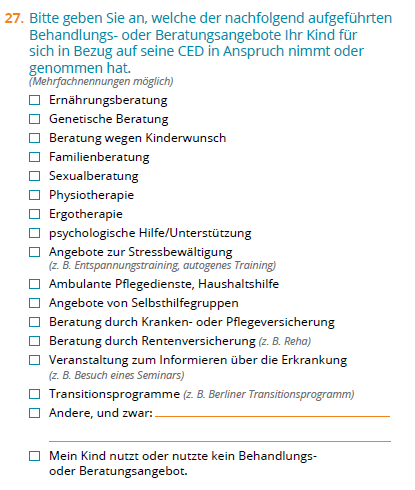
Please indicate which of the following treatment or counselling services your child has used or has used in relation to their CED. (Multiple selections are possible.)
- Nutritional counselling
- Genetic counselling
- Counselling for family planning
- Family counselling
- Sexual counselling
- Physiotherapy
- Occupational therapy
- Psychological help/support
- Stress management programs (e.g., relaxation training, autogenic training)
- Outpatient nursing services, home help
- Support group offerings
- Counselling from health or long-term care insurance
- Counselling from pension insurance (e.g., rehabilitation)
- Information events about the condition (e.g., attending a seminar)
- Transition programs (e.g., Berlin Transition Program)
- Other, please specify: …
- My child does not use or has not used any treatment or counselling services.


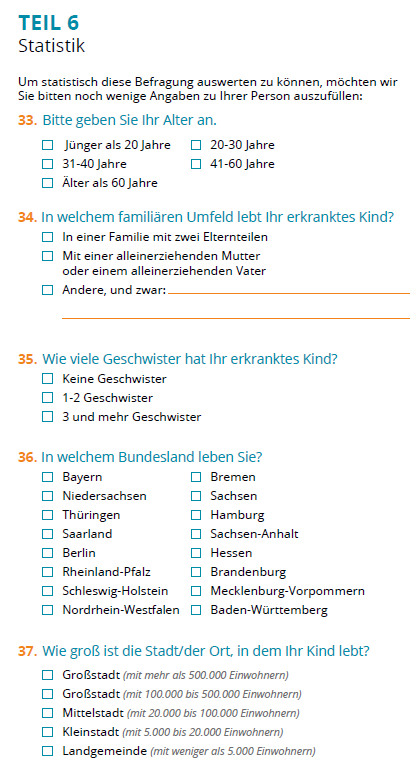


PART 6 Statistics

To help us analyse this survey statistically, please provide a few details about yourself:

1. Please indicate your age.

- Younger than 20 years
- 20-30 years
- 31-40 years
- 41-60 years
- Older than 60 years

1. In what family environment does your ill child live?

- In a family with two parents
- With a single mother or single father
- Other, please specify:

1. How many siblings does your ill child have?

- No siblings
- 1-2 siblings
- 3 or more siblings

1. In which federal state (Bundesland) do you live?

- Bavaria
- Bremen
- Lower Saxony
- Saxony
- Thuringia
- Hamburg
- Saarland
- Saxony-Anhalt
- Berlin
- Hesse
- Rhineland-Palatinate
- Brandenburg
- Schleswig-Holstein
- Mecklenburg-Western Pomerania
- North Rhine-Westphalia
- Baden-Württemberg

1. How large is the city/town where your child lives?

- Major city (with more than 500,000 residents)
- Major city (with 100,000 to 500,000 residents)
- Mid-sized city (with 20,000 to 100,000 residents)
- Small town (with 5,000 to 20,000 residents)
- Rural area (with fewer than 5,000 residents)

1.
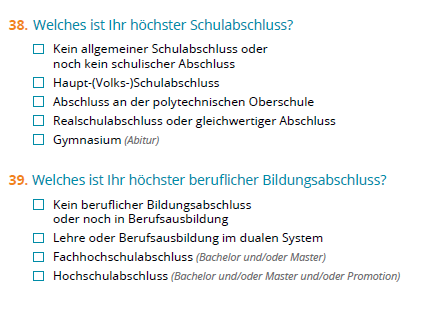
What is your highest level of education?

- No general school degree or no educational qualification
- Primary school completion
- Completion of a polytechnic school
- Secondary school diploma or equivalent
- Gymnasium (Abitur)

1. What is your highest vocational education level?

- No vocational education or still in vocational training
- Completed apprenticeship or vocational training in the dual system
- Bachelor's and/or Master's degree from a university of applied sciences (Fachhochschule)
- Bachelor's and/or Master's and/or PhD from a university
