# Supplementary material for: Healthcare service use in paediatric inflammatory bowel disease: a questionnaire on patient and parent care experiences in Germany
Source: BMC Gastroenterol. 2023 Nov 6;23:378. doi: 10.1186/s12876-023-03021-w (PMC10626645; doi:10.1186/s12876-023-03021-w)
Supplement: Supplementary file 2 [file 12876_2023_3021_MOESM2_ESM.docx]

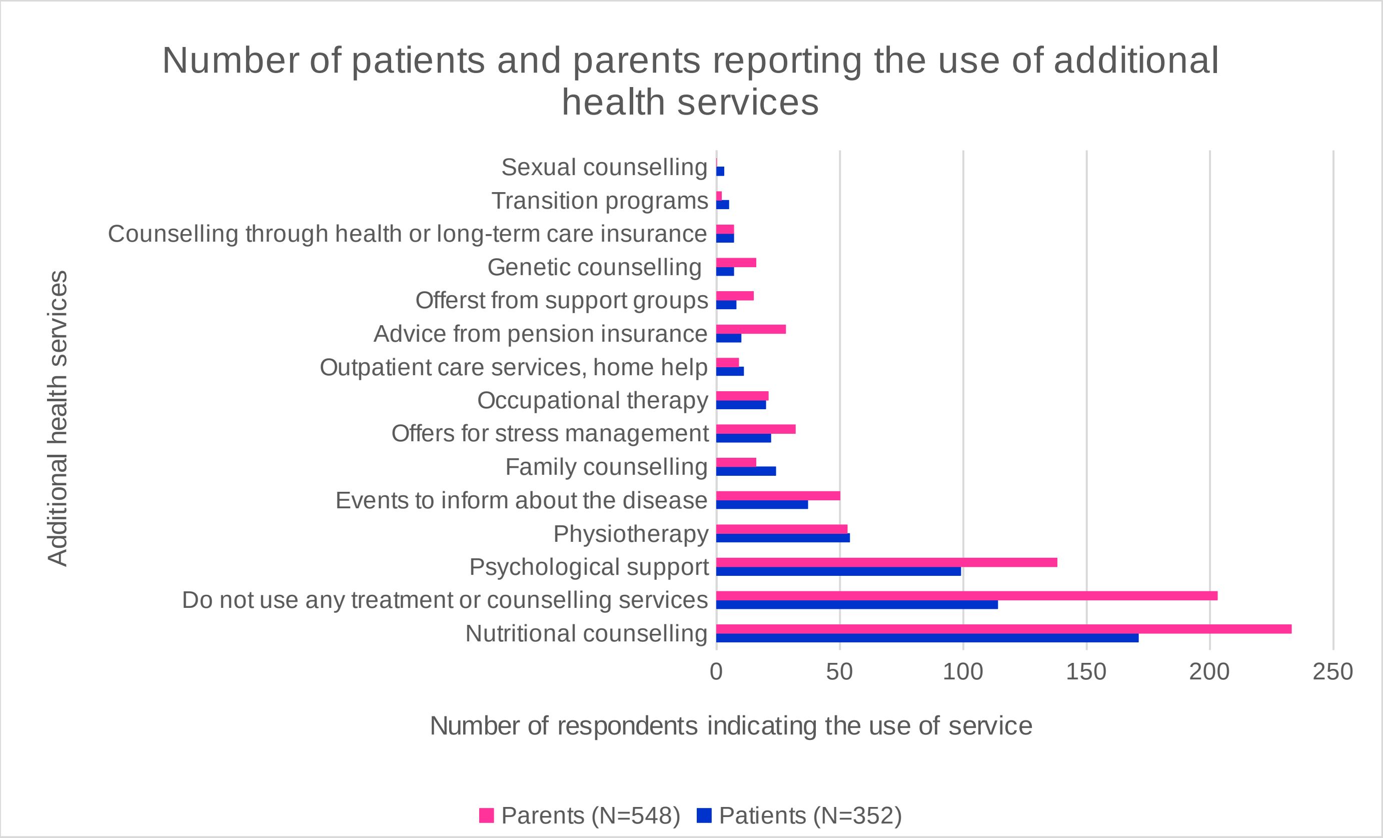

Number of patients and parents reporting the use of additional health services
Additional health services
Sexual counselling
Transition programs
Counselling through health or long-term care insurance
Genetic counselling
Offerst from support groups
Advice from pension insurance
Outpatient care services, home help
Occupational therapy
Offers for stress management
Family counselling
Events to inform about the disease
Physiotherapy
Psychological support
Do not use any treatment or counselling services
Nutritional counselling
0
50
100
150
200
250
Number of respondents indicating the use of service
Parents (N=548)
Patients (N=352)
